# Supplementary material for: Stochastic ordering of complexoform protein assembly by genetic circuits
Source: PLoS Comput Biol. 2020 Jun 29;16(6):e1007997. doi: 10.1371/journal.pcbi.1007997 (PMC7351240; doi:10.1371/journal.pcbi.1007997)
Supplement: S1 Appendix — (PDF) [file pcbi.1007997.s001.pdf]

S1 Appendix: Genetic circuits

Each of the four genetic circuits producing proteins *X* and *Y* (parallel, cascade, series uncoupled, and series coupled) are implemented as a set of reactions describing transcription, translation, protein export, and protein binding, as outlined below for each circuit. The following abbreviations are used in this appendix:

P:	Promoter
RNAP:	RNA polymerase
RNAPgene:	RNA polymerase bound to DNA, in RNA elongation phase
mrna:	Transcribed mRNA sequence
Rib:	Ribosome
X / *Y:*	Protein *X* / *Y* (in the cellular interior)
X.out / *Y.out:*	Protein *X* / *Y* (exterior to the cell)
sca.sca:	Scaffold protein, with two unoccupied binding sites
X/Y.sca:	Scaffold protein, with one binding site occupied by protein *X* / *Y*
X.X:	Scaffold protein, occupied by two *X* proteins (termed *XX*)
X.Y:	Scaffold protein, occupied by an *X* protein and a *Y* protein (termed *XY*)
Y.Y:	Scaffold protein, occupied by two *Y* proteins (termed *YY*)
PRO:	Second polymerase co-generated with protein *X* in the cascade genetic circuit
Ø:	Used to indicate loss of a reactant

Parallel Genetic Circuit

<u>Transcription</u>

$$P + RNAP \underset{k_{pro-}}{\overset{k_{pro}}{\rightleftarrows}} P.RNAP \xrightarrow{k_{gene}} P + RNAPgene1$$

$$RNAPgene1 \xrightarrow{k_m} mrna1 + RNAP$$

$$RNAPgene1 \xrightarrow{k_m} mrna2 + RNAP$$

<u>Translation</u>

$$mrna1 + Rib \xrightarrow{k_p} mrna1 + Rib + X$$

$$mrna2 + Rib \xrightarrow{k_p} mrna2 + Rib + Y$$

$$mrna1 \xrightarrow{k_{mrna-loss}} \emptyset$$

$$mrna2 \xrightarrow{k_{mrna-loss}} \emptyset$$

<u>Protein export / binding</u>

$$X \xrightarrow{k_{out}} X.out$$

$$Y \xrightarrow{k_{out}} Y.out$$

$$X.out + sca.sca \xrightarrow{k_{bind}} X.sca$$

$$Y.out + sca.sca \xrightarrow{k_{bind}} Y.sca$$

$$X.sca + X.out \xrightarrow{k_{bind}} X.X$$

$$X.sca + Y.out \xrightarrow{k_{bind}} X.Y$$

$$Y.sca + X.out \xrightarrow{k_{bind}} X.Y$$

$$Y.sca + Y.out \xrightarrow{k_{bind}} Y.Y$$

$$X.out \xrightarrow{k_{out-loss}} \emptyset$$

$$Y.out \xrightarrow{k_{out-loss}} \emptyset$$

Cascade Genetic Circuit

Transcription

$$P1 + RNAP \underset{k_{pro-}}{\overset{k_{pro}}{\rightleftharpoons}} PT7 \xrightarrow{k_{gene}} P1 + RNAPgene1$$

$$RNAPgene1 \xrightarrow{k_m} mrna1 + RNAP$$

$$P2 + PRO \underset{k_{pro-}}{\overset{k_{pro}}{\rightleftharpoons}} PPRO \xrightarrow{k_{gene}} P2 + PROgene2$$

$$PROgene2 \xrightarrow{k_m} mrna2 + PRO$$

$$PRO \xrightarrow{k_{pro-loss}} \emptyset$$

Translation

$$mrna1 + Rib \xrightarrow{k_p} mrna1 + Rib + X + PRO$$

$$mrna2 + Rib \xrightarrow{k_p} mrna2 + Rib + Y$$

$$mrna1 \xrightarrow{k_{mrna-loss}} \emptyset$$

$$mrna2 \xrightarrow{k_{mrna-loss}} \emptyset$$

Protein export / binding

$$X \xrightarrow{k_{out}} X.out$$

$$Y \xrightarrow{k_{out}} Y.out$$

$$X.out + sca.sca \xrightarrow{k_{bind}} X.sca$$

$$Y.out + sca.sca \xrightarrow{k_{bind}} Y.sca$$

$$X.sca + X.out \xrightarrow{k_{bind}} X.X$$

$$X.sca + Y.out \xrightarrow{k_{bind}} X.Y$$

$$Y.sca + X.out \xrightarrow{k_{bind}} X.Y$$

$$Y.sca + Y.out \xrightarrow{k_{bind}} Y.Y$$

$$X.out \xrightarrow{k_{out-loss}} \emptyset$$

$$Y.out \xrightarrow{k_{out-loss}} \emptyset$$

Series Uncoupled Genetic Circuit

<u>*Transcription*</u>

$$P + RNAP \underset{k_{pro-}}{\overset{k_{pro}}{\rightleftarrows}} P.RNAP \xrightarrow{k_{gene}} P + RNAPgene1$$

$$RNAPgene1 \xrightarrow{k_m} mrna1 + RNAPgene2$$

$$RNAPgene2 \xrightarrow{k_m} mrna2 + RNAP$$

<u>*Translation*</u>

$$mrna1 + Rib \xrightarrow{k_p} mrna1 + Rib + X$$

$$mrna2 + Rib \xrightarrow{k_p} mrna2 + Rib + Y$$

$$mrna1 \xrightarrow{k_{mrna-loss}} \emptyset$$

$$mrna2 \xrightarrow{k_{mrna-loss}} \emptyset$$

<u>*Export / binding*</u>

$$X \xrightarrow{k_{out}} X.out$$

$$Y \xrightarrow{k_{out}} Y.out$$

$$X.out + sca.sca \xrightarrow{k_{bind}} X.sca$$

$$Y.out + sca.sca \xrightarrow{k_{bind}} Y.sca$$

$$X.sca + X.out \xrightarrow{k_{bind}} X.X$$

$$X.sca + Y.out \xrightarrow{k_{bind}} X.Y$$

$$Y.sca + X.out \xrightarrow{k_{bind}} X.Y$$

$$Y.sca + Y.out \xrightarrow{k_{bind}} Y.Y$$

$$X.out \xrightarrow{k_{out-loss}} \emptyset$$

$$Y.out \xrightarrow{k_{out-loss}} \emptyset$$

Series Coupled Genetic Circuit

Transcription

$$P + RNAP \underset{k_{pro-}}{\overset{k_{pro}}{\rightleftarrows}} P.RNAP \xrightarrow{k_{gene}} P + RNAPgene1$$

$$RNAPgene1 \xrightarrow{k_m} mrna1 + RNAPgene2$$

$$RNAPgene2 \xrightarrow{k_m} mrna2 + RNAP$$

Translation

$$mrna1 + Rib \xrightarrow{k_p} mrna1 + Rib.mrna1 + X$$

$$mrna2 + Rib.mrna1 \xrightarrow{k_p} mrna2 + Rib + Y$$

$$mrna1 \xrightarrow{k_{mrna-loss}} \emptyset$$

$$mrna2 \xrightarrow{k_{mrna-loss}} \emptyset$$

Protein export / binding

$$X \xrightarrow{k_{out}} X.out$$

$$Y \xrightarrow{k_{out}} Y.out$$

$$X.out + sca.sca \xrightarrow{k_{bind}} X.sca$$

$$Y.out + sca.sca \xrightarrow{k_{bind}} Y.sca$$

$$X.sca + X.out \xrightarrow{k_{bind}} X.X$$

$$X.sca + Y.out \xrightarrow{k_{bind}} X.Y$$

$$Y.sca + X.out \xrightarrow{k_{bind}} X.Y$$

$$Y.sca + Y.out \xrightarrow{k_{bind}} Y.Y$$

$$X.out \xrightarrow{k_{out-loss}} \emptyset$$

$$Y.out \xrightarrow{k_{out-loss}} \emptyset$$
