# Supplementary material for: Stochastic ordering of complexoform protein assembly by genetic circuits
Source: PLoS Comput Biol. 2020 Jun 29;16(6):e1007997. doi: 10.1371/journal.pcbi.1007997 (PMC7351240; doi:10.1371/journal.pcbi.1007997)
Supplement: S2 Appendix — (PDF) [file pcbi.1007997.s002.pdf]

S2 Appendix. Simulation rates and initial conditions

Every simulation is initiated with an amount of promoter, RNA polymerase, and ribosome, as well as 10 unoccupied scaffold proteins with two binding sites each. In the simulations, the translation rate k_p, the protein export rate k_{out}, and the protein binding rate k_{bind} are all varied individually. Unless otherwise indicated, the initial conditions and rates are as indicated below for both the stochastic and deterministic computational solutions. All units are indicated in arbitrary units of concentration, [C], and time, t.

Parallel / Series Uncoupled / Series Coupled Genetic Circuits

Reactant	Initial value [C]
P	2
RNAP	100
Rib	100
sca.sca	10

Rate	Value
k_{pro}	1 $[C]^{-1}t^{-1}$
k_{pro-}	0.01 t^{-1}
k_{gene}	0.1 t^{-1}
k_m	1 t^{-1}
k_p	1 $[C]^{-1}t^{-1}$
$k_{mrna-loss}$	0.2 t^{-1}
k_{out}	1 t^{-1}
k_{bind}	1 $[C]^{-1}t^{-1}$
$k_{out-loss}$	0.01 t^{-1}

Cascade Genetic Circuit

Reactant	Initial value [C]
P1	2
P2	2
RNAP	100
Rib	100
sca.sca	10

Rate	Value
k_{pro}	1 $[C]^{-1}t^{-1}$
k_{pro-}	0.01 t^{-1}
$k_{pro-loss}$	0.01 t^{-1}
k_{gene}	0.1 t^{-1}
k_{m}	1 t^{-1}
k_{p}	1 $[C]^{-1}t^{-1}$
$k_{mrna-loss}$	0.2 t^{-1}
k_{out}	1 t^{-1}
k_{bind}	1 $[C]^{-1}t^{-1}$
$k_{out-loss}$	0.01 t^{-1}
