# Supplementary material for: Stochastic ordering of complexoform protein assembly by genetic circuits
Source: PLoS Comput Biol. 2020 Jun 29;16(6):e1007997. doi: 10.1371/journal.pcbi.1007997 (PMC7351240; doi:10.1371/journal.pcbi.1007997)

S3 Appendix: mRNA degradation rates

To investigate the role of mRNA degradation in regulating the final distribution of the complexoform, we conducted simulations of the series-uncoupled circuit with the same parameters as outlined in S2 Appendix. But we varied the mRNA degradation rate constant, $k_{mrna-loss}$. Our results show that the rate constant of mRNA degradation does affect the distribution of the complexoform. The outcome is equivalent to varying the translation rate constant. For example, increasing the rate constant of mRNA degradation is equivalent to decreasing the rate constant of translation. It has the overall effect of slowing down the process of complexoform assembly. The results for the series-uncoupled circuit are summarized in the figure below, in which both the stochastic solutions (*XX*: black circles; *YY*: white circles; *XY*: grey circles) and deterministic solutions (*XX*: solid black line; *YY*: dashed line; *XY*: solid grey line) are shown.

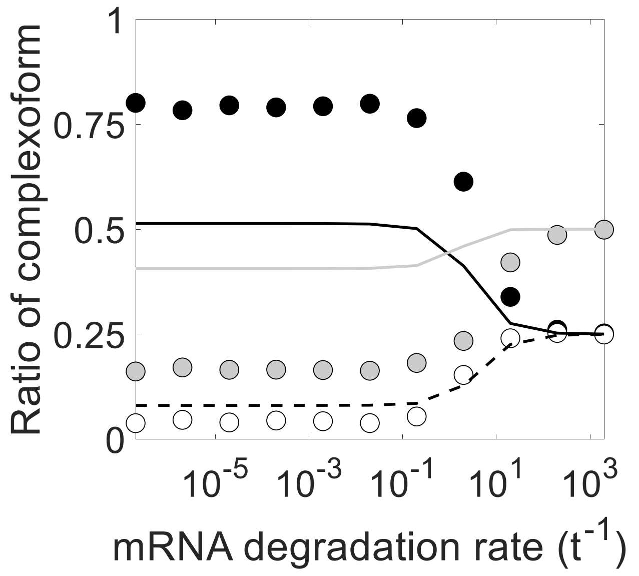

Supplement: S3 Appendix — (PDF) [file pcbi.1007997.s003.pdf]
