# Supplementary material for: Stochastic ordering of complexoform protein assembly by genetic circuits
Source: PLoS Comput Biol. 2020 Jun 29;16(6):e1007997. doi: 10.1371/journal.pcbi.1007997 (PMC7351240; doi:10.1371/journal.pcbi.1007997)

S4 Appendix: Promoter binding rates

The first process in each simulation is the binding between a promoter and the RNA polymerase. To demonstrate the role of this initial step in the final distribution of the complexoform, we run stochastic and deterministic simulations for each of the four genetic circuits (parallel, cascade, series uncoupled, and series coupled) using the rate constants summarized in S2 Appendix while varying the promoter binding constant. The results are summarized in the figure below for both the stochastic solutions (*XX*: black circles; *YY*: white circles; *XY*: grey circles) and deterministic solutions (*XX*: solid black line; *YY*: dashed line; *XY*: solid grey line). Changing the promoter binding constant does affect the time to assembly (not shown in the figure) and, therefore, alters the expected complexoform distribution from the deterministic solutions. However, the promoter binding constant does not affect the stochastic simulations because the rate of the downstream reactions and genetic circuit architecture dominate.

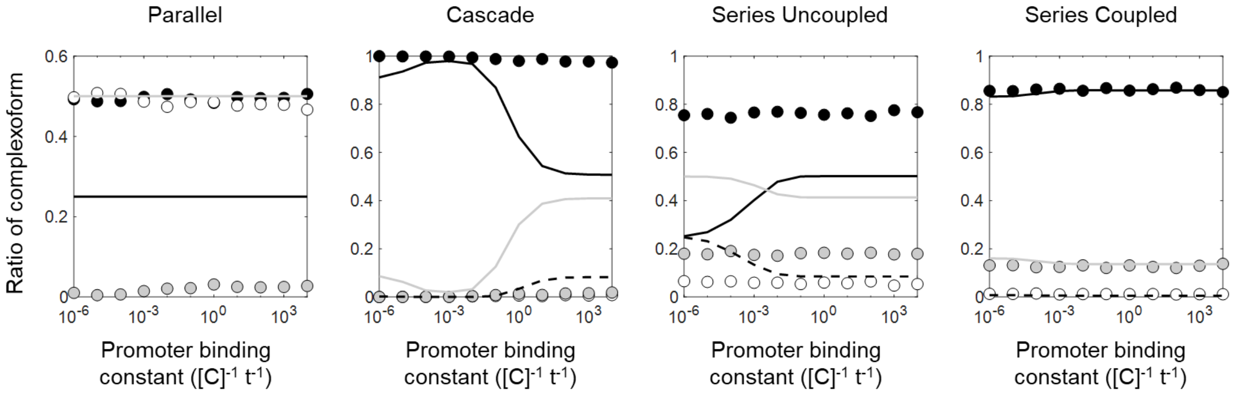

Supplement: S4 Appendix — (PDF) [file pcbi.1007997.s004.pdf]
