# Supplementary material for: Stochastic ordering of complexoform protein assembly by genetic circuits
Source: PLoS Comput Biol. 2020 Jun 29;16(6):e1007997. doi: 10.1371/journal.pcbi.1007997 (PMC7351240; doi:10.1371/journal.pcbi.1007997)
Supplement: S5 Appendix — (PDF) [file pcbi.1007997.s005.pdf]

S5 Appendix: Dependence of Complexoform Distribution on Temporal Profiles of External Protein Concentrations

In this work, we model two proteins, X and Y. They undergo protein expression, export to the extracellular medium, and bind to a limited number of external scaffoldin binding sites. In all computational simulations, the final distribution of the complexoform is determined by both the genetic circuit architecture and the kinetic rate constants. Ultimately, the external protein concentrations determine the amount of scaffoldin-bound proteins according to:

$$\frac{d[X.sca]}{dt} = k_{bind} \cdot [X.out] \cdot [sca]$$

and

$$\frac{d[Y.sca]}{dt} = k_{bind} \cdot [Y.out] \cdot [sca]$$

Here, $[X.sca]$ and $[Y.sca]$ denote a scaffoldin binding site occupied by protein X or Y, respectively, $[X.out]$ and $[Y.out]$ denote the concentration of external protein, $[sca]$ denotes the available (i.e., unoccupied) scaffoldin binding sites, and k_{bind} is the binding rate constant of protein X or Y binding to scaffoldin. Thus, the final distribution of the complexoform is not determined solely by the final external concentrations of the proteins, but by the cumulative external protein concentrations up until the time of assembly (T_{asb}) according to the integrated rate equation, i.e.:

$$[X.sca] = k_{bind} \int_0^{T_{\mathrm{asb}}} [X.out] \cdot [sca] \, dt$$

and

$$[Y.sca] = k_{bind} \int_0^{T_{\mathrm{asb}}} [Y.out] \cdot [sca]\, dt$$

The external protein concentrations are determined by all upstream effects (i.e., the rate constants of preceding processes, as well as the genetic circuit architecture). Thus, the variable [*X.sca*] or [*Y.sca*] alone does not completely describe the distribution of the complexoform. Nonetheless, the final ratio of protein X and protein Y in the complexoform is directly related to the cumulative external protein concentrations.

To illustrate this, we calculate the cumulative external protein concentrations in our stochastic simulations, multiplied by the available scaffoldin binding sites over time, as:

$$[X_{cumulative}] = k_{bind} \int_0^{T_{\mathrm{asb}}} [X.out] \cdot [sca]\, dt$$

and

$$[Y_{cumulative}] = k_{bind} \int_0^{T_{\mathrm{asb}}} [Y.out] \cdot [sca]\, dt$$

We determine the normalized ratio of protein X and Y as:

$$[X_{cumulative,normalized}] \equiv \frac{[X_{cumulative}]}{[X_{cumulative}] + [Y_{cumulative}]}$$

and

$$[Y_{cumulative,normalized}] \equiv \frac{[Y_{cumulative}]}{[X_{cumulative}] + [Y_{cumulative}]}$$

In the stochastic simulations, we compare this metric to the observed normalized ratio of protein X and Y in the distribution of the complexoform, starting from 20 available binding sites (i.e., 10

scaffold proteins with two binding sites each). We simulate each condition to compare the cumulative external protein concentrations to the distribution of the complexoform for each condition (as in Figure 3). The results are summarized for 1,000 stochastic simulations in the figure below using the rates summarized in S2 Appendix. The figures show the average fraction of X in the complexoform (solid circles) and the cumulative normalized fraction of X in the cell exterior (open circles), as well as the average discrepancy between the two over each set of 1,000 simulations (solid lines).

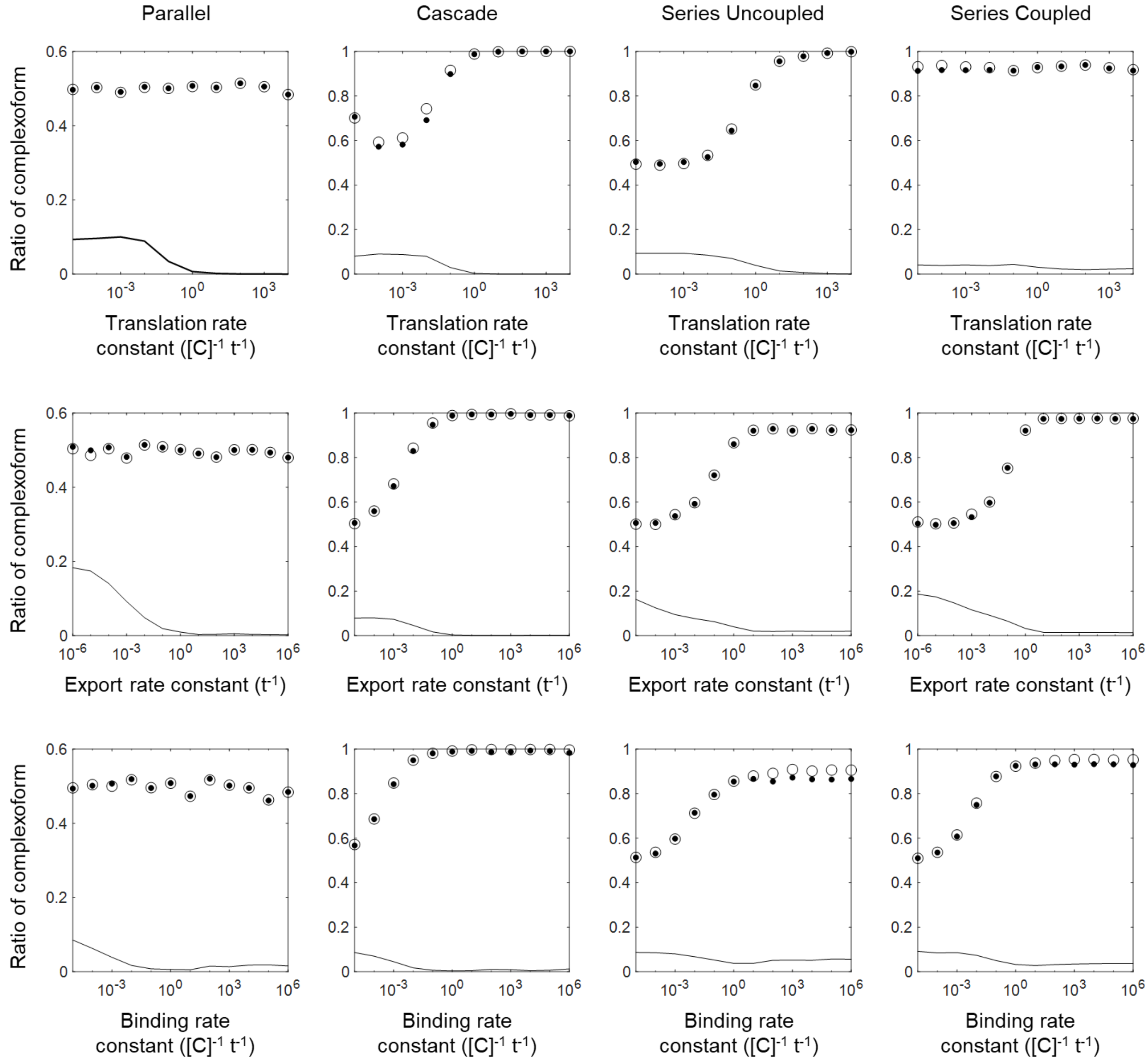

Here, we do not distinguish between scaffold proteins with two X or two Y bound and scaffold proteins with one X and one Y. Instead, we count the full distribution of the proteins in the complexoform. Our results show that for all conditions, the averages over the 1,000 simulations for the complexoform distribution match the cumulative external protein concentrations, clearly indicating that the complexoform distribution is determined by the cumulative external protein concentration.

In summary, the cumulative external protein concentrations (which is in turn determined by upstream effects of both the genetic circuit architecture and the kinetic rate constants of preceding steps) govern the average complexoform distribution. However, we note that the stochastic nature of protein binding can cause the complexoform distribution to deviate from the cumulative external protein concentrations.
