# Supplementary material for: Stochastic ordering of complexoform protein assembly by genetic circuits
Source: PLoS Comput Biol. 2020 Jun 29;16(6):e1007997. doi: 10.1371/journal.pcbi.1007997 (PMC7351240; doi:10.1371/journal.pcbi.1007997)
Supplement: Attachment — Submitted filename: response _ plos comp bio _ 0416.pdf [file pcbi.1007997.s007.pdf]

UNIVERSITY OF CALIFORNIA, DAVIS

BERKELEY • DAVIS • IRVINE • LOS ANGELES • MERCED • RIVERSIDE • SAN DIEGO • SAN FRANCISCO SANTA BARBARA • SANTA CRUZ

Cheemeng Tan
Associate Professor, Department of Biomedical Engineering
One Shields Ave, Davis, CA 95616

Phone: 530-752-7849
Fax: 530-754-5739
email: cmtan@ucdavis.edu

Apr 23, 2020

Dear Editor,

Please find the attached revised manuscript, "Stochastic ordering of complexoform protein assembly by genetic circuits" for publication in PLoS Computational Biology.

We greatly appreciate the feedback with regards to our manuscript. To address all reviewer comments, we have conducted additional simulations. The new results are included in the revised manuscript (S3-S5 Appendix). We kept all changes tracked within the main text (highlighted in red). The point-to-point response (in black) is as follows:

Editorial notes

In addition to the reviewer comments below, please also consider my own comment that this work seems to be related to earlier work linked to here -
- https://journals.plos.org/plosone/article?id=10.1371/journal.pone.0032032 -- which also used a simulation-based approach to determine distribution of complexoforms and their kinetics. If so, it may be worth citing and discussing.

We thank the editor for the suggestion. We have now discussed the prior publication in the introduction (Line 64).

"Furthermore, a recent computational study has studied the formation of protein complexes using existing data on protein-protein interaction networks. This prior work shows that the composition of a protein complex can drift over time even when the simulation starts from the same initial condition. The work suggests that other cellular mechanisms must exist to prevent the compositional drift of some protein complexes."

Figure Files:

While revising your submission, please upload your figure files to the Preflight Analysis and Conversion Engine (PACE) digital diagnostic tool, https://pacev2.apexcovantage.com. PACE

helps ensure that figures meet PLOS requirements. To use PACE, you must first register as a user. Then, login and navigate to the UPLOAD tab, where you will find detailed instructions on how to use the tool. If you encounter any issues or have any questions when using PACE, please email us at figures@plos.org.

We have prepared all the main figures in the high-resolution eps format.

Data Requirements:

Please note that, as a condition of publication, PLOS' data policy requires that you make available all data used to draw the conclusions outlined in your manuscript. Data must be deposited in an appropriate repository, included within the body of the manuscript, or uploaded as supporting information. This includes all numerical values that were used to generate graphs, histograms etc.. For an example in PLOS Biology see here: http://www.plosbiology.org/article/info%3Adoi%2F10.1371%2Fjournal.pbio.1001908#s5.

A supporting document, *S6 – Data.xlsx*, has been created containing the data for all figures in both the main text and the other supplementary figures. The data for each figure is included on a separate tab.

Reviewer #1

This is a nice study elucidating the role of biological circuitry in control and assembly of complexoform proteins. With a few modifications, it should be accepted for publication. Please take the following suggestions into account:

• The authors should take into account the effect of different promoter strengths and different ribosome binding site strengths for each separate gene. The authors should analyze the effect of changing the promoter and RBS strengths for an individual gene, and compare this to the effect of changing the genetic circuit architecture. If circuit architecture effects tend to dominate over promoter and RBS binding site effects, this should be explained.

We thank the reviewer for the suggestion. We have conducted additional simulations by varying the promoter binding strengths for all four genetic modules (parallel, cascade, series uncoupled, and series coupled). We find that changing the promoter binding constant does affect the time to assembly and, therefore, alters the complexoform distribution predicted from deterministic solutions. However, the promoter binding constant does not affect the stochastic simulation outcomes because the downstream kinetic rate constants and genetic circuit architecture dominate the outcome. These results are summarized in the figure below, showing the stochastic solutions (XX: black circles; YY: white circles; XY: grey circles) and deterministic solutions (XX: solid black line; YY: dashed line; XY: solid grey line). These results are now included in the S4 Appendix.

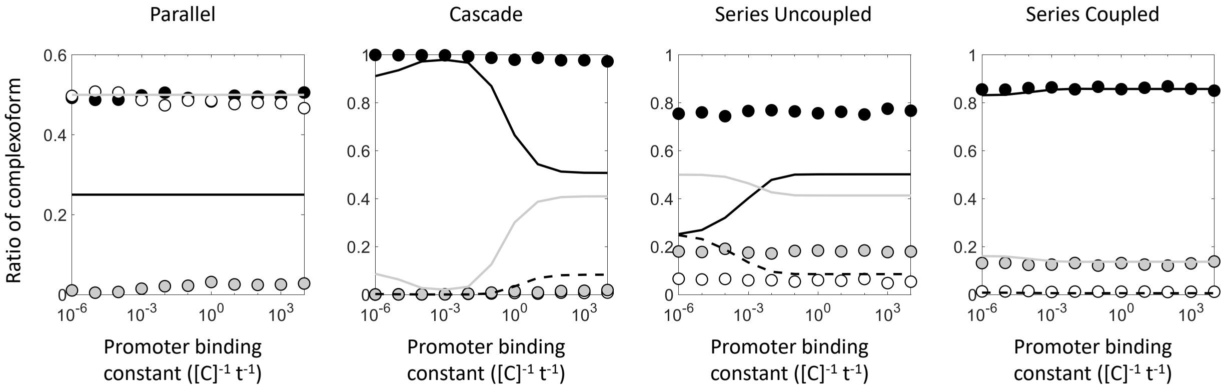

• The authors should include some discussion of the relative abundance of each type of circuit architecture found in natural genomes, for assembling known complexoforms. Is there a particular circuit layout which tends to appear more often than the others?

We thank the reviewer for the suggestion. This suggestion is intriguing and may lead to a separate paper like the original network motif paper by Uri Alon (Milo, Ron, et al. "Network motifs: simple building blocks of complex networks." *Science* 298.5594 (2002): 824-827.). However, it is a serious undertaking that deviates drastically from the focus of our work/messages.

Our work claims that specific arrangements of genetic modules can lead to compositional differences in complexoforms. Our prior submission included a brief discussion on the genetic modules that exist in nature (Line 413-426). The analysis of circuit frequency, however, is challenging because there is no existing database that specifically relates the genome sequence to complexoforms. Furthermore, to perform a systematic investigation, one will have to analyze genome sequences of several species to find the codon. This analysis is nontrivial

due to the unknown/unannotated promoter and transcription start sites in many genome sequences. In addition, we will have to match the genome sequence to the protein-protein interaction maps. Therefore, while we are interested in such analysis, it is likely beyond the scope of this work. We have included a brief discussion of the future direction (Line 429).

• The authors should discuss their work in relation to the following literature:
o Yeung et al. Biophysical Constraints Arising from Compositional Context in Synthetic Gene Networks. Cell Systems. 2017.

o In particular, the authors should include a discussion of how the orientation of genetic circuits in a parallel configuration affects transcription, and how this might impact their model.

We thank the reviewer for the suggestion. We have added a brief discussion in Line 383.

“First, our work did not consider the details of how genetic context affects the transcription or translation rate of genes. For instance, a prior work [20] shows that the arrangement of two genes can affect the level, dynamic range, and sensitivity of their expression. The spatial arrangement of two genes will affect the expression of our parallel genetic module. Specifically, if the parallel genetic module is inducible by certain chemicals, its sensitivity and dynamic range of induction would also be affected by the arrangement of the two genes, as suggested by the prior work. In our work, we have not considered the induction dynamics of the genetic module, which may serve as another regulatory force of complexoforms.”

• The term “complexoform” is of limited use in the literature (a Google Scholar search returns four relevant results). Accordingly, in order to assure clarity, the authors should introduce the term as “complex isoforms” as is done in Fonslow et al.

We thank the reviewer for the suggestion. We have added alternative terms of complexoform in the abstract and introduction.

Reviewer #2

This is an interesting and novel study, where computational simulations are used to analyze a recently appreciated phenomenon -- complexoforms. In particular, the authors thoroughly analyzed how gene circuit architecture and rate constants can affect distribution of complexoforms. They have also thoroughly compared deterministic and stochastic simulations to examine the potential contribution of noise in this dynamics.

The study is well designed and the paper is well written and easy to follow. The model formulation and subsequent analysis are fairly standard. But the key innovation of the study is the application context (to a novel problem, as far as I can tell). The work defines a useful blueprint for the dynamic analysis of complexoforms. The modeling framework (in particular) and the results can be useful for guiding future experimental analysis.

I am in support of the publication of the work with some minor clarifications and revisions, as detailed below.

Specific points:
1. In essence, the different circuit architectures dictate the temporal dynamics of X and Y, which in turn bind the the different scaffold sites. As such, it is not entirely surprising that different architectures would modulate the find distribution of complexoforms differently. I wonder if it is possible to deduce some more general rules that govern the final distribution based on the temporal profiles of X and Y (outside the cell).

We thank the reviewer for the suggestion. We have conducted stochastic simulations for all four genetic modules (parallel, cascade, series uncoupled, and series couples) by varying the translation rate constant, export rate constant, and binding rate constant. For each of the conditions, we monitored the abundance of protein *X* and *Y* in the eventual complexoform distribution with a total of 10 dimers (i.e., 20 available binding sites) for each simulation. We formulated a new metric, termed the cumulative external protein concentration, based on the integrated rate equation for protein binding to scaffoldin, and correlated this metric to the final complexoform distributions. We find that this metric predicts the average complexoform distribution, but that the stochastic nature of the protein binding causes some deviation for each simulation. The results are summarized in the figure below, showing the average fraction of X in the complexoform (solid circles) and the cumulative normalized fraction of X in the cell exterior (open circles), as well as the average discrepancy between the two (solid lines). The new results are included in the new S5 Appendix.

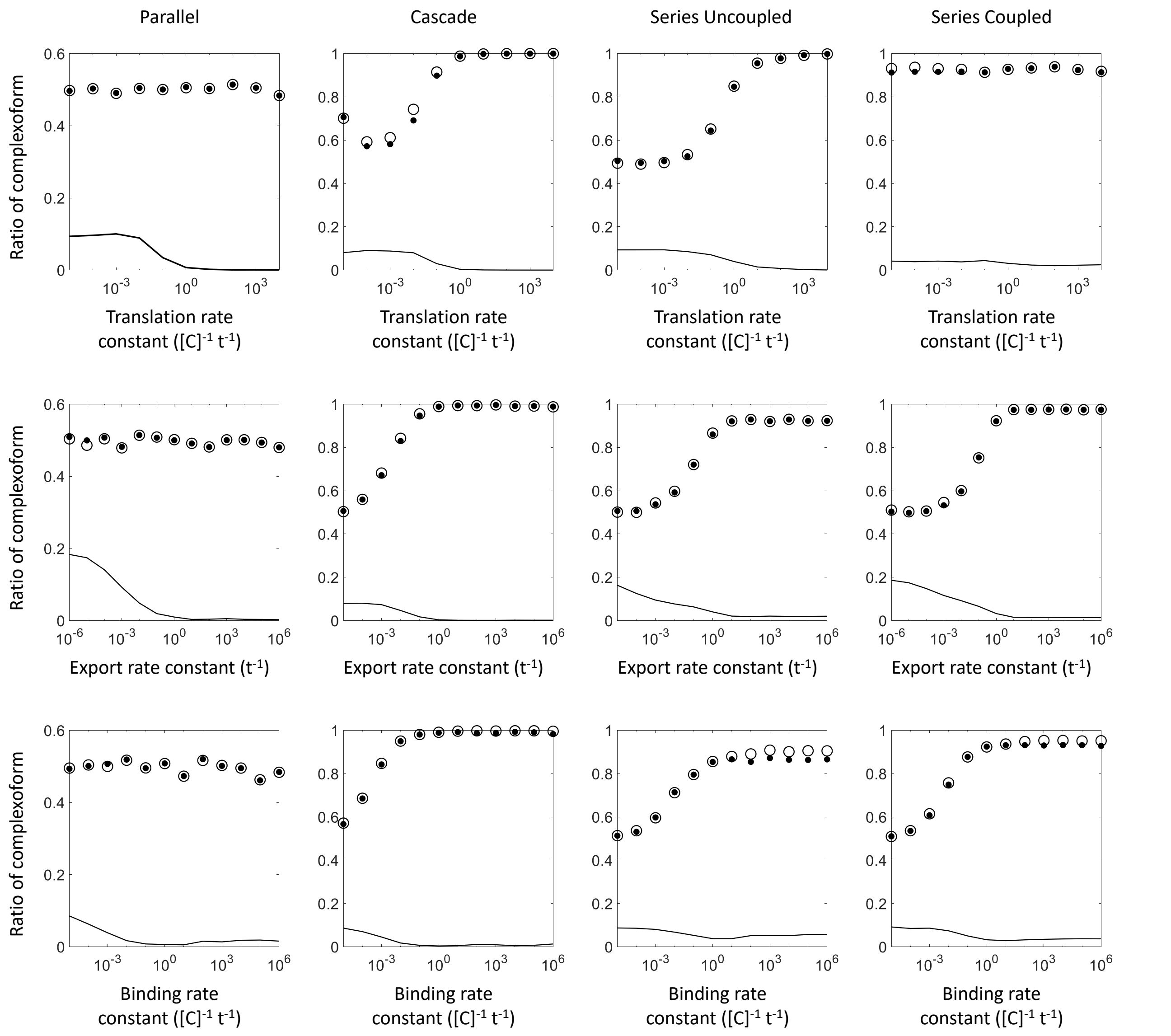

2. Related to the point above, the binding kinetics of X and Y to scaffold site should be important as well. If I understand it correctly, the authors assume the binding to be irreversible (Table 1). I wonder what happens to the distributions if the binding is reversible, which appears to be more plausible.

We have clarified this point starting in Line 557 of the revised manuscript:

"Although data shows a slow dissociation of the dockerin/cohesin complex, we assume that the binding is irreversible to allow us to study the structure of the completed assembly right after protein expression, transport, and assembly. We note that with a slowly reversible binding, the complexoform distribution should eventually equilibrate with the external solution concentrations of protein in solution over long time scales."

3. Does the number of binding sites matter? At it stands, the choice of the number seems ad hoc.

We thank the reviewer for raising this point and have clarified this point in the revised manuscript. As long as the number of binding sites available is relatively small in number, the effects described in the manuscript apply. We have clarified this point starting in Line 516 of the revised manuscript:

"Although the exact cellulosome complex can contain many types of catalytic modules and scaffoldin binding sites [5], we adopt a model in which the external scaffold has 10 available sites to which two different proteins can directly bind, as this model captures the competing role of both timing and gene circuit architecture in modulating the complexoform." In other words, the key for the conclusions to be valid is that the number of binding sites be appreciably small, and similar conclusions could have been reached with, say, 5 or 20 scaffoldin binding sites instead of 10.

4. There was a lot of focus on comparing deterministic modeling and stochastic modeling. The resulting insights are not well articulated and need to be clarified in a revision. Is there experimental data that the modeling results can be compared to?

We thank the reviewer for the suggestion. We have now included a brief discussion (Line 392) about our insight for the comparison of deterministic and stochastic solutions.

"Second, one challenge in our work is to obtain the analytical solution of the stochastic equations. For instance, previous work by Laurenzi, Renyi, Mcquarrie, and Ishida [21-24] have solved the analytical solutions for A+B↔C. To obtain the solution, the authors exploit the conservation of mass between A, B, and C. Finding similar analytical solutions to our model involves two challenges. First, there are multiple bimolecular reactions in our model. Second, it is challenging to incorporate the conservation of mass for intermediate reactions of our model. There is, however, qualitative agreement between our simulations and prior analytical solution. For instance, Laurenzi shows that stochastic solution deviates from a deterministic solution when the number of molecules is low. We observe this trend generally for all our results because the molecular number in our system is low. Furthermore, Laurenzi shows that the variance of molecular concentrations peaks at intermediate reaction time when the reaction becomes irreversible. Likewise, we observe that when reaction rate becomes slower, our system takes longer time to approach the steady-state solution. The longer transient kinetics increase the duration when the system exhibits high variance of molecular concentration. This enhanced variance likely leads to the significant deviation between the stochastic and deterministic solutions when reaction rate constants are low."

Reviewer #3

In the manuscript titled " Stochastic ordering of complexoform protein assembly by genetic circuits", Jensen et al. used combination of stochastic simulation and deterministic differential equation model to investigate how parameters such as timing of transcription, translation, and transport rate impact formation preferences of complexoform. They found that in two different extreme scenarios, complexoform variations are also vastly different. At the end, the authors used a real biological example to verify their framework. I think the authors did a good job explaining this relatively new topic and made the text fairly easy to follow. Discussing a relatively less studied new area, this work used standard simulation methods to shed some new light on possible mechanisms controlling complexoform preferences, attributing some of the control to gene circuit layout. This finding hopefully would inspire more future work in this area to investigate this interesting and important problem. I found the manuscript to be informative, the approaches used appropriate, and would recommend for its publication given my following concerns/comments to be addressed:

1. One major concern of mine is the lack of any analytical results. Numerical simulations are definitely preferred in many scenarios, including this work. But careful analysis often is also needed. It would complement numerical simulations well and often offer a fuller picture. I understand analytical results are not possible for complex systems, but in this case, there is no feedback and an analysis of the simplified scenarios should be within reach.

We thank the reviewer for the suggestion. Analytical solutions for bimolecular reactions (A+B<->C) were published by Laurenzi, Renyi, Mcquarrie, Ishida. The exact solution exploits the conservation of mass between species A, B, and C. However, extending such solution methods to our model was not possible for at least two reasons 1) our model involves multiple bimolecular reactions 2) for intermediate reactions, exploiting the conservation of mass will introduce significant estimation error. Due to these reasons, we did not present analytical solutions for our work because that would involve significant effort and lie outside the focus of the work.

Instead, we have now included a brief discussion (Line 392) about analytical solutions, and the qualitative consistency between our and the prior work.

“Second, one challenge in our work is to obtain the analytical solution of the stochastic equations. For instance, previous work by Laurenzi, Renyi, Mcquarrie, and Ishida [21-24] have solved the analytical solutions for A+B↔C. To obtain the solution, the authors exploit the conservation of mass between A, B, and C. Finding similar analytical solutions to our model involves two challenges. First, there are multiple bimolecular reactions in our model. Second, it is challenging to incorporate the conservation of mass for intermediate reactions of our model. There is, however, qualitative agreement between our simulations and prior analytical solution. For instance, Laurenzi shows that stochastic solution deviates from a deterministic solution when the number of molecules is low. We observe this trend generally for all our results because the molecular number in our system is low. Furthermore, Laurenzi shows that the variance of molecular concentrations peaks at intermediate reaction time when the reaction becomes irreversible. Likewise, we observe that when reaction rate becomes slower, our system takes longer time to approach the steady-state solution. The longer transient kinetics increase the duration when the system exhibits high variance of molecular concentration. This enhanced variance likely leads to the significant deviation between the stochastic and deterministic solutions when reaction rate constants are low.”

2. I am curious why the authors did not consider mRNA degradation as one key parameters. mRNA degradation has been shown to be a key regulatory mechanism in biology.

We thank the reviewer for the suggestion. We have conducted additional simulations by varying the mRNA degradation rate for the series-uncoupled genetic module. We find that the mRNA degradation rate does affect the complexoform distribution, and that the outcome is equivalent to varying the translation rate constant: Increasing the rate constant of mRNA degradation is equivalent to decreasing the rate constant of translation, and has the overall effect of slowing down the process of complexoform assembly. The results for the series uncoupled module are summarized in the figure below, in which both the stochastic solutions (XX: black circles; YY: white circles; XY: grey circles) and deterministic solutions (XX: solid black line; YY: dashed line; XY: solid grey line) are shown. The new results are included in the new S3 Appendix.

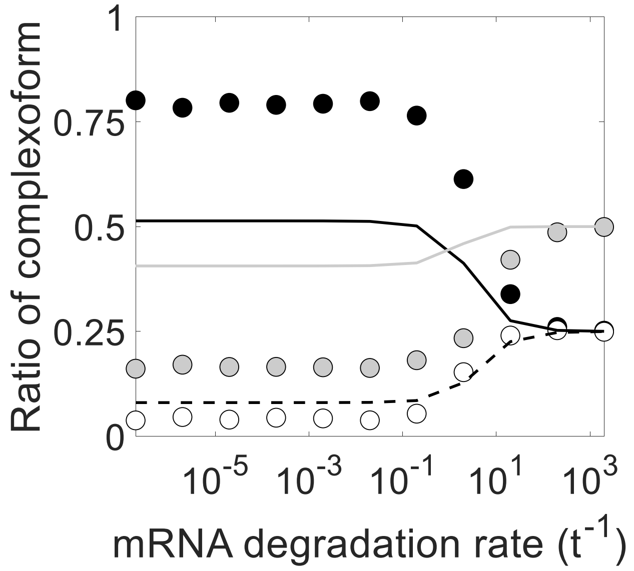

In addition, recent work (Wu et al, Cell Systems 6, 206-215, 2018) showed that in the series coupled scenarios, the order of the gene X and Y would affect their stability and hence could cause a change in the complexoform formation biases.

We thank the reviewer for the suggestion. We have added a brief discussion in Line 408.

"Third, the arrangement of two genes in the series coupled module may affect the expression level of the genes. Typically, the second gene is expressed at a lower level than the first gene. A prior work [25] has quantified the effect of GC content, size, and folding energy of the first gene on the expression of the second gene. Incorporating these details will improve the prediction power of our model on the distribution of complexoforms."
